# Efficacy of erector spinae plane block for postoperative analgesia after liver surgeries: a systematic review and meta-analysis

**DOI:** 10.1186/s12871-024-02635-1

**Published:** 2024-07-20

**Authors:** Jiajia Qian, Xueqin Wang

**Affiliations:** Day surgery ward, Huzhou Central Hospital, Affiliated Central Hospital of HuZhou University, 1558 Sanhuan North Road, Huzhou, Zhejiang Province China

**Keywords:** Regional analgesia, Pain, Hepatectomy, Hepatic surgery, Anesthesia

## Abstract

**Background:**

Data on the effectiveness of erector spinae plane block (ESPB) for patients undergoing liver surgeries is limited and inconclusive. We hereby aimed to systematically review if ESPB can provide adequate analgesia after liver surgery.

**Methods:**

PubMed, CENTRAL, Scopus, Embase, and gray literature were examined up to 25th April 2023 for randomized controlled trials (RCTs) comparing ESPB with control or spinal analgesia.

**Results:**

Nine RCTs were included of which three compared ESPB with spinal analgesia. 24-hour opioid consumption did not differ significantly between ESPB vs. control (MD: -35.25 95% CI: -77.01, 6.52 I^2^ = 99%) or ESPB vs. spinal analgesia (MD: 2.32 95% CI: -6.12, 10.77 I^2^ = 91%). Comparing pain scores between ESPB and control, a small but significant effect favoring ESPB was noted at 12 h and 48 h, but not at 6–8 h and 24 h. Pain scores did not differ between ESPB and spinal analgesia. The risk of postoperative nausea and vomiting was also not significantly different between ESPB vs. control or spinal analgesia. GRADE assessment shows moderate certainty of evidence.

**Conclusion:**

ESPB may not provide any significant postoperative analgesia in liver surgery patients. There was a tendency of reduced opioid consumption with ESPB. Limited data also showed that ESPB and spinal analgesia had no difference in pain scores and 24-hour analgesic consumption.

**Supplementary Information:**

The online version contains supplementary material available at 10.1186/s12871-024-02635-1.

## Introduction

Patients undergoing liver surgeries experience considerable postoperative pain which requires optimal management to improve patient satisfaction. Despite the availability of minimally invasive surgical techniques, improved technology, and a wide array of analgesic options, pain control after liver surgeries remains a challenge and there has been a constant effort to improve outcomes and enhance the quality of recovery [1]. The concept of enhanced recovery after surgery has been successfully implemented in the case of liver surgeries and an important component of the program is the use of multimodal analgesia and reduced dependence on opioids [2]. Indeed, opioids are central to pain control after most abdominal surgical procedures and are associated with significant adverse events like nausea, vomiting, sedation, constipation, and respiratory depression [3]. Given such side effects and the probability of long-term dependence with the use of opioids, there is a need for efficient and easy-to-administer regional nerve blocks which can reduce pain scores with minimal adverse events.

The erector spinae plane block (ESPB) is one such regional anesthetic modality that has become widely popular since its introduction in 2016 [4]. The technique consists of the injection of local anesthetic agents between the erector spinal muscles and the thoracic transverse processes targeting the dorsal-ventral rami of the spinal nerves and sympathetic ganglia by spreading craniocaudal and into the paravertebral region [5]. Given that the ESPB has a wide compartment, the absorption of the injectate is rapid and results in a higher bioavailability as compared to other blocks [6]. Furthermore, the wide extent of the erector spinae muscle allows for injections at different levels resulting in analgesic effects in different regions. A meta-analysis of 13 RCTs has found ESPB to provide better analgesia with reduced postoperative opioid consumption in patients undergoing breast surgeries [7]. Likewise, Koo et al [8] in a pooled analysis of 17 RCTs found ESPB to have a significantly better analgesic effect in comparison with no block in thoracic surgeries. Recently, Viderman et al [9] combined data from studies on different abdominal surgical procedures to find that ESPB reduced opioid requirement but had no difference in pain scores as compared to no block. Since abdominal surgery can involve a lot of different procedures each with different risks and pain levels, the efficacy of ESPB must be tested for more specific regions. Earlier, Bhushan et al [10] attempted to examine the efficacy of ESPB for liver surgeries but could include only six trials and compared ESPB with different control groups. To generate more homogenous and updated evidence, we hereby conducted this review to examine the analgesic efficacy of ESPB compared to no block or spinal analgesia in patients undergoing liver surgeries.

## Materials and methods

### Search

A review protocol was prepared and registered on the directory PROSPERO (CRD42023414636). An experienced medical librarian along with one of the reviewers were involved in the literature search which included the electronic databases of PubMed, CENTRAL, Scopus, and Embase. To ensure completeness of the search, we also included gray literature via Google Scholar and Open Gray (http://www.opengrey.eu). www.clinicaltrials.gov. The search concluded on 25th April 2023. The reviewers used the keywords: “erector spinae plane block”, “hepatic”, “hepatectomy”, “abdominal surgery”, and “liver surgery”. A common search strategy was devised for all databases (Supplementary Table 1). The medical librarian and the reviewer examined all results without language restriction and deduplicated them using a reference manager software (EndNote). Two reviewers then proceeded with study screening initially by titles/abstracts and then by full-texts of relevant studies. All decisions on study selection were taken by consensus. The search was supplemented by a direct search of references of eligible studies.

### Eligibility

We included RCTs conducted on a *Population* of adult liver surgery patients. Patients in the study group received an *Intervention* of ESPB at any perioperative time. Patients in the *Comparison* group received no/sham block or spinal analgesia. The study reported any of the following *Outcomes*: Pain values, total analgesic consumption after surgery, or postoperative nausea and vomiting (PONV). We excluded studies with overlapping data, retrospective studies, and not exclusively on liver surgery patients.

### Data extraction

Last author, publication year, study location, type of liver surgery, the anesthetic agent used, level of ESPB, puncture location, control group details, sample size, method of verification of ESPB, patient-controlled analgesia (PCA), type of rescue analgesia and other analgesics, and outcome data were extracted using a pre-formatted table by two reviewers. For missing data, the corresponding author of the article was contacted once by email. The primary outcome was total opioid consumption in 24 h in intravenous morphine equivalents. Secondary outcomes were pain measured on a 10-point scale at 6–8 h, 12 h, 24, and 48 h and PONV.

Quality assessment of studies was conducted by two reviewers using the Cochrane Collaboration risk of bias-2 tool [11]. Every section of the tool is then marked as low risk, high risk, or some concerns based on the flowchart provided. Grading of Recommendations Assessment, Development, and Evaluation (GRADE) tool based on the GRADEpro GDT software was used to judge the certainty of the evidence.

### Statistical analysis

“Review Manager” (RevMan, version 5.3; Nordic Cochrane Centre [Cochrane Collaboration], Copenhagen, Denmark; 2014) was the software for the meta-analysis. Data on pain and 24-hour opioid consumption was extracted as mean and standard deviation (SD) for the meta-analysis. If the included studies reported data as median and range or interquartile values, it was changed to mean and SD by the formula of Wan et al [12]. Data provided in graphs was converted into numbers by Engauge Digitizer Version 12.1. All data on opioid consumption was standardized to morphine equivalents utilizing a standardized converter [13]. Total opioid consumption and pain outcomes were combined as mean difference (MD) with 95% confidence intervals (CI) in a random-effects model. PONV data were pooled to generate risk ratios (RR). The I^2^ statistic in the meta-analysis evaluated inter-study heterogeneity with values > 50% considered as substantial heterogeneity. Data for ESPB vs. control and ESPB vs. spinal analgesia was pooled separately. The review conformed to the PRISMA reporting guidelines [14].

## Results

### Search

**Fig. 1:**
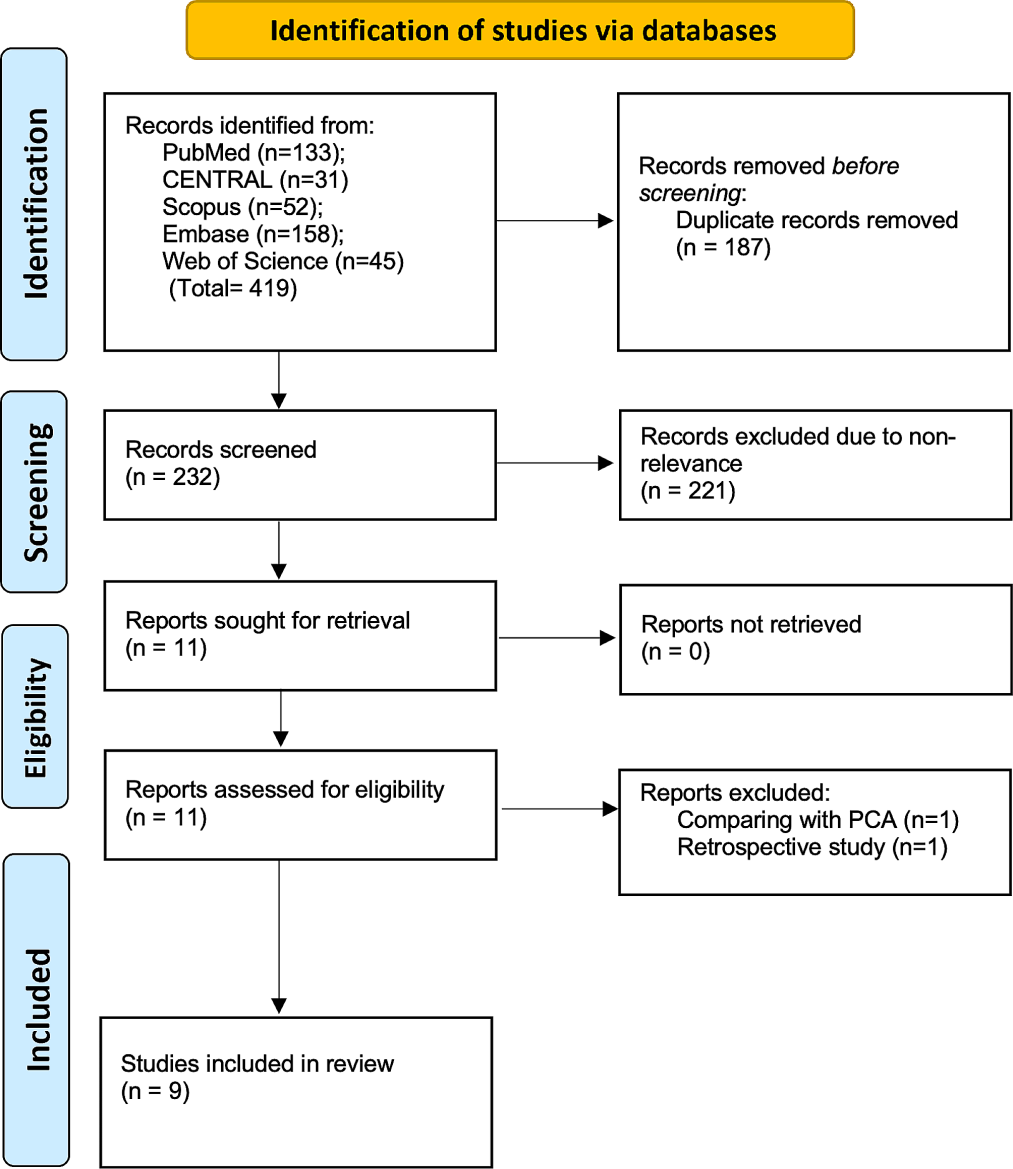
Study flow chart

419 articles were found based on the search strategy. All duplicates were removed and 232 articles were identified. On initial screening, 221 were excluded. Eleven studies underwent full-text analysis and nine were included [15–23] (Fig. 1).

### Study details

Data extracted from the studies is shown in Table 1. The studies were published between 2019 and 2022 and were from South Korea, China, Egypt, Turkey, and Pakistan. All studies were on liver resection while one was on percutaneous radiofrequency ablation. All studies used ultrasound guidance and administered bilateral blocks. In two trials, continuous ESPB blocks were administered. The levels of the block ranged from T7 to T9. The type, dosage, and concentration of local anesthetics were variable among studies. Lidocaine, ropivacaine, and bupivacaine were the anesthetic agents used. In two studies, dexmedetomidine was injected with the anesthetic agent. In three trials, spinal analgesia was administered in the control group. Two trials used intrathecal morphine while one used epidural analgesia. The sample size per group ranged from 20 to 30. Most studies did not report on the method of verification of ESPB. The drugs used in PCA were fentanyl, morphine, sufentanil, and tramadol.

**Table 1:** Details of studies included in meta-analysis

Study	Location	Surgical procedure	ESPB method	Anesthetic agent	Puncture location	Control group	Sample size	Verification of ESPB	PCA	Other analgesics
Kang 2019 [23]	South Korea	Living donor laparoscopic hepatectomy	USG guided bilateral single injection	40 ml 0.5% ropivacaine	T8	Intra-thecal morphine 400 µg	ESPB: 27 Control: 27	NR	Fentanyl	IV Ibuprofen 400 mg every 6 h
Fu 2020 [22]	China	Partial hepatectomy	USG guided bilateral single injection	40 ml 0.5% ropivacaine	T8	No block	ESPB: 30 Control: 30	NR	None	Rescue analgesic as IV morphine 5 mg; if no relief then IV fentanyl 25–50 µg
Mostafa 2020 [20]	Egypt	Percutaneous radiofrequency ablation	USG guided bilateral single injection	10 ml 2% lidocaine and 10 ml 0.5% bupivacaine	T7	Sham block	ESPB: 30 Control: 30	NR	None	Rescue analgesic as IV morphine 2 mg
Kang 2021 [21]	South Korea	Living donor laparoscopic hepatectomy	USG guided bilateral programmed intermittent bolus injection every 3 h for 48 h	40 ml 0.5% ropivacaine	T8	Intra-thecal morphine	ESPB: 29 Control: 30	NR	Fentanyl	Rescue analgesic as IV meperidine 25 mg; if no relief IV hydromorphone was given
Kim 2021 [15]	South Korea	Laparoscopic liver resection	USG guided bilateral single injection	40 ml 0.5% ropivacaine	T9	No block	ESPB: 35 Control: 35	NR	Fentanyl	IV Ibuprofen 400 mg every 6 h; Rescue analgesic as IV hydromorphone
Elshafie 2022 [19]	Egypt	Liver resection	USG guided bilateral single injection	40 ml 0.25% bupivacaine with dexmedetomidine	T7	No block	ESPB: 20 Control: 20	NR	None	Rescue analgesic as IV fentanyl bolus 0.5 µg/kg
Hacıbeyoğlu 2022 [17]	Turkey	Hepatectomy	USG guided bilateral single injection	40 ml 0.375% bupivacaine with dexmedetomidine	T8	No block	ESPB: 25 Control: 25	Loss of hot-cold sensation below and above the bilateral T8 dermatome level	Morphine	Not reported
Huang 2022 [18]	China	Laparoscopic hepatectomy	USG guided bilateral single injection	30 ml 0.5% ropivacaine	T8	No block	ESPB: 25 Control: 25	Not assessed	Sufentanil & tramadol	Not reported
Zubair 2022 [16]	Pakistan	Living donor hepatectomy	USG guided bilateral continuous injection	Continuous 0.125% bupivacaine 10-12 ml/h	T7-8	Continuous epidural analgesia with 0.125% bupivacaine 10-12 ml/h	ESPB: 20 Control: 20	NR	None	Rescue analgesic as IV Nalbuphine; IV paracetamol 1 g as standard

USG, ultrasound; ESPB, erector spinae plane block; PCA, patient controlled analgesia; IV, intravenous; h, hours; T, thoracic; NR, not reported

### Meta-analysis

**Fig. 2:**
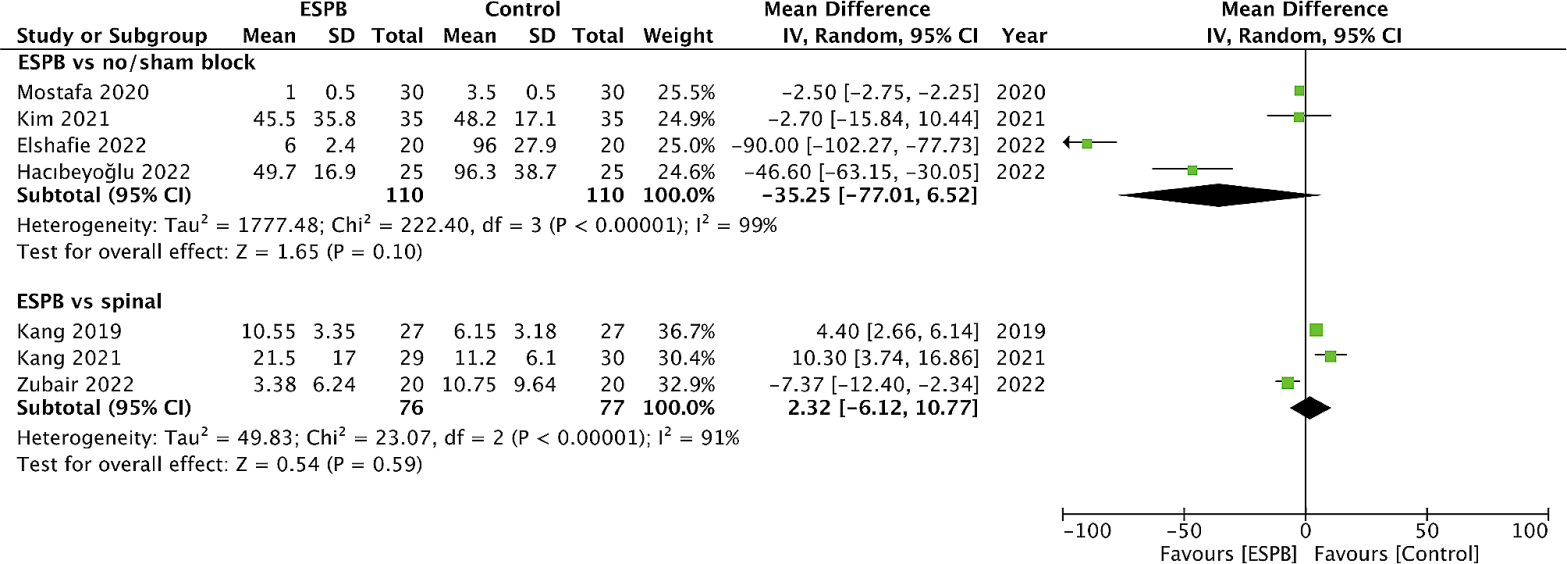
Meta-analysis of 24-hour total opioid consumption between ESPB vs. control and spinal analgesia

24-hour opioid consumption was reported in seven trials. Pooled analysis showed that there was a tendency of lower opioid consumption with ESPB as compared to the control group, however, the results were statistically non-significant (MD: -35.25 95% CI: -77.01, 6.52 I^2^ = 99%) (Fig. 2). However, there was no difference in 24-hour opioid consumption between ESPN and spinal analgesia (MD: 2.32 95% CI: -6.12, 10.77 I^2^ = 91%) (Fig. 2).

**Fig. 3:**
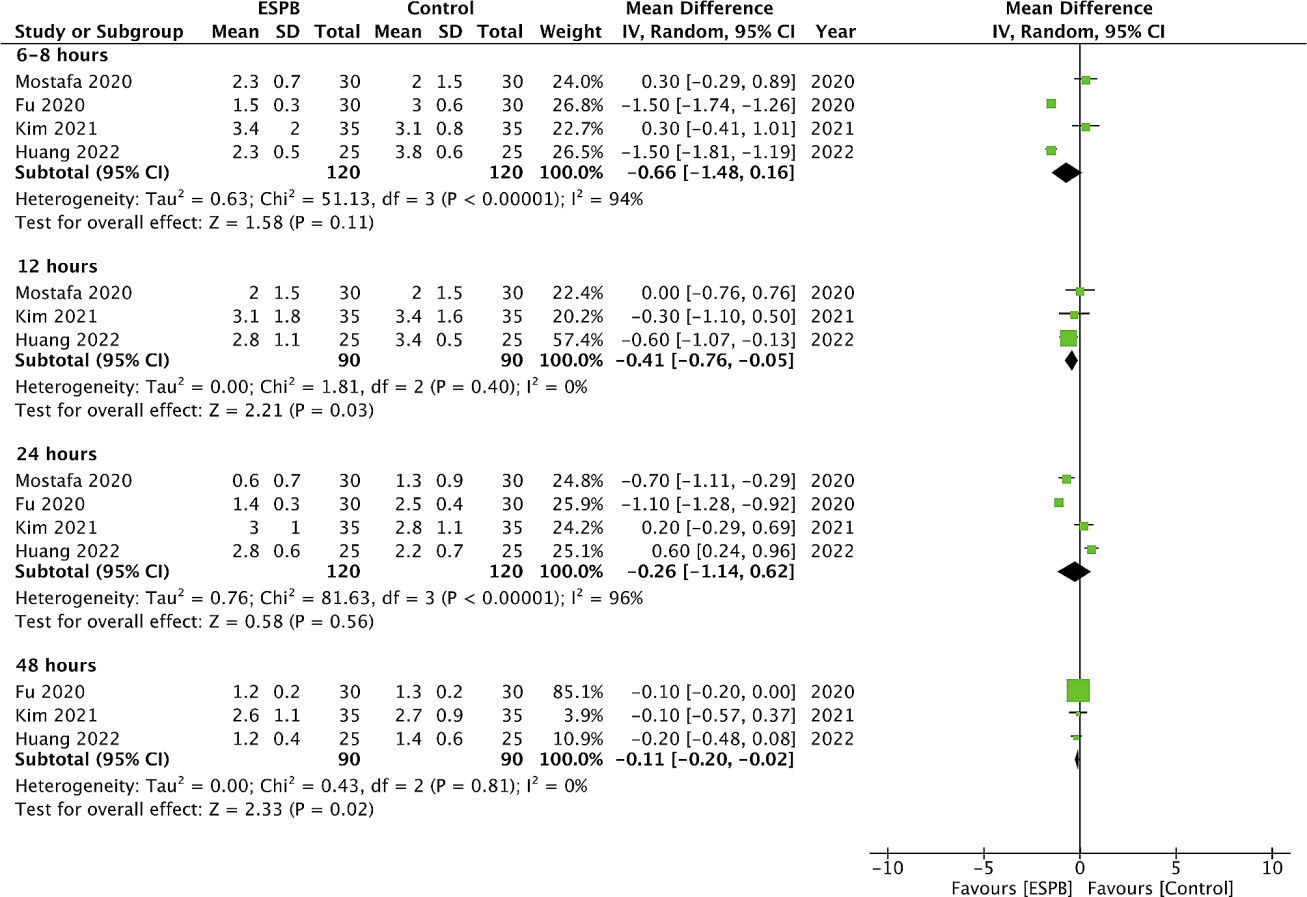
Meta-analysis of pain scores between ESPB vs. control

**Fig. 4:**
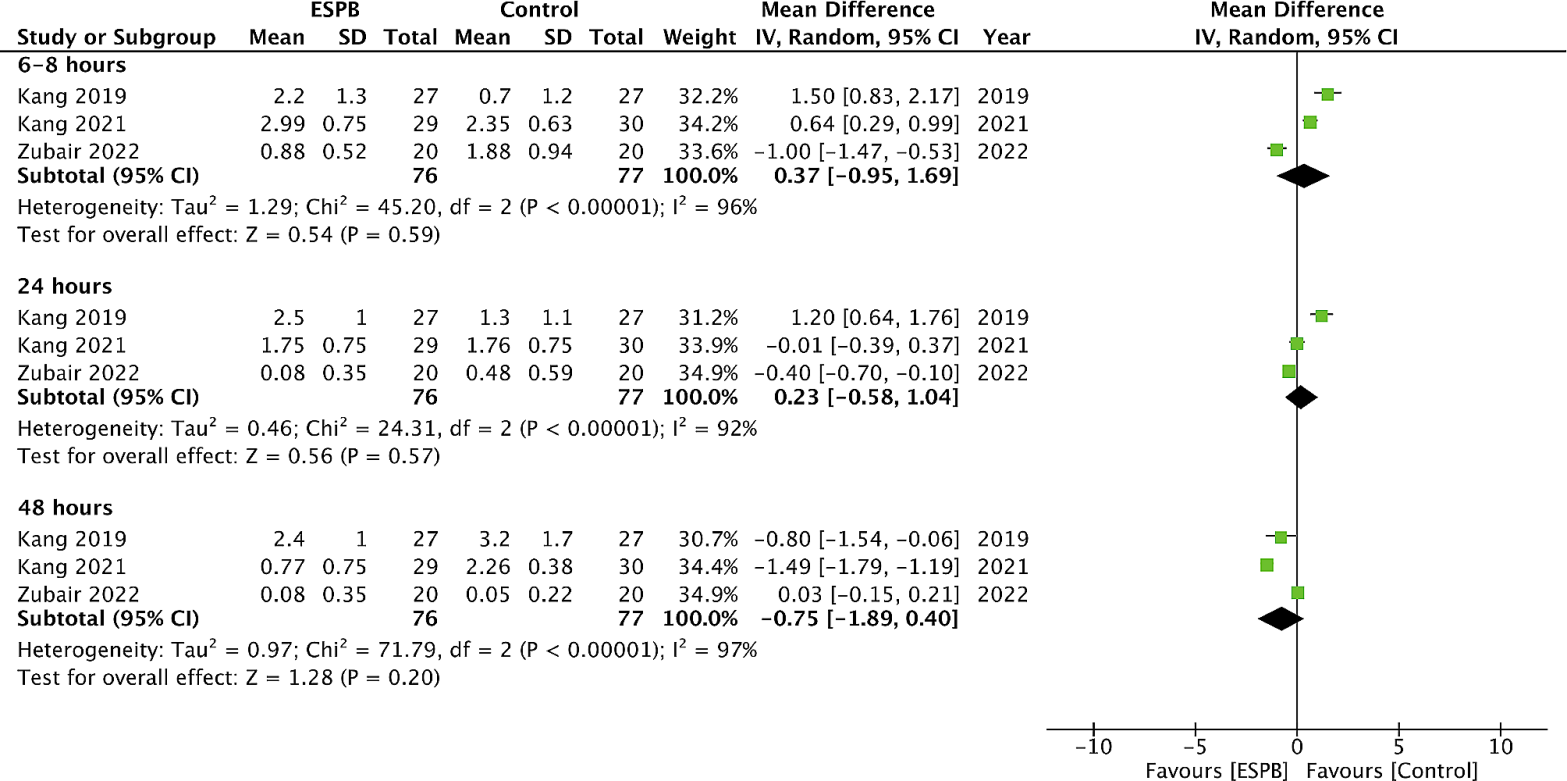
Meta-analysis of pain scores between ESPB vs. spinal analgesia

Comparing pain scores between ESPB and control groups, we noted no statistically significant difference at 6–8 h (MD: -0.66 95% CI: -1.48, 0.16 I^2^ = 94%) and 24 h (MD: -0.26 95% CI: -1.14, 0.62 I^2^ = 96%). However, a small but significant effect favoring ESPB was noted at 12 h (MD: -0.41 95% CI: -0.76, -0.05 I^2^ = 0%) and 48 h (MD: -0.11 95% CI: -0.20, -0.02 I^2^ = 0%) (Fig. 3). On the other hand, the meta-analysis failed to demonstrate any significant difference in pain scores between ESPB and spinal analgesia at 6–8 h (MD: 0.37 95% CI: -0.95, 1.69 I^2^ = 96%), 24 h (MD: 0.23 95% CI: -0.58, 1.04 I^2^ = 92%) and 48 h (MD: -0.75 95% CI: -1.89, 0.40 I^2^ = 97%) (Fig. 4).

**Fig. 5:**
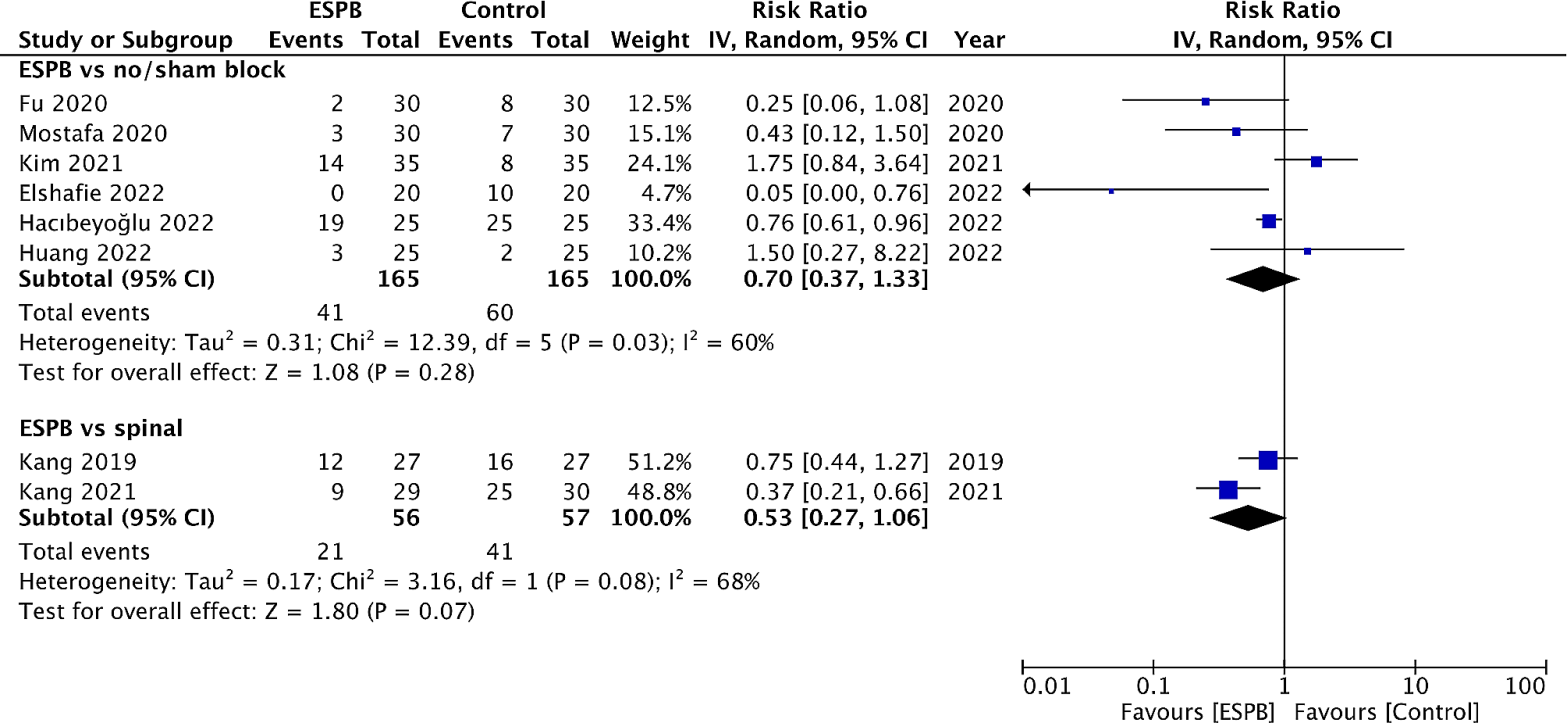
Meta-analysis of PONV between ESPB vs. control and spinal analgesia

Eight studies reported data on PONV. Meta-analysis showed no significant difference in the risk of POV between ESPB and control groups (RR: 0.70 95% CI: 0.37, 1.33 I^2^ = 60%) (Fig. 5). Similarly, a meta-analysis of just two studies showed no difference in the risk of POV between ESPB and spinal analgesia (RR: 0.53 95% CI: 0.27, 1.06 I^2^ = 68%) (Fig. 5).

### Risk of bias and GRADE assessment

All, except for two trials, were high quality and had a low overall risk of bias (Supplementary Table 2). The study of Fu et al [22] had a high risk of bias while Mostafa et al [20] had some concerns. GRADE assessment of evidence is shown in Supplementary Table 3. The certainty was moderate for all outcomes.

## Discussion

In recent years, regional interfascial blocks have expanded the scope of regional anesthesia providing postoperative analgesia in a variety of surgical interventions. In comparison with the peripheral nerve block wherein a local anesthetic agent is injected around a singular nerve or group of nerves, the injectate for regional interfascial blocks is placed between fascial layers thereby anesthetizing all nerves transversing along the tissue plane as well as adjacent areas [24]. Amongst the several popular blocks used routinely in clinical practice is the ESPB, developed quite recently in 2016 by Forero et al [4]. While it was initially described by the authors for the management of thoracic neuropathic pain, its usage has expanded greatly with anesthetists reporting its use for breast [7], thoracic [8], and spinal surgeries [25]. Since the anesthetic agent in ESPB is deposited beneath the erector spinae muscle near the tip of the transverse process of the vertebrae [26], and the muscle itself transverses the entire span of the spinal cord, it is postulated that the level of injection can have different analgesic effects and can be used for a variety of surgical procedures. In the current review, we investigated the efficacy of ESPB for pain control after liver surgeries by pooling data from nine RCTs.

Opioids constitute the primary drugs that are used in the management of postoperative pain in most surgical procedures. Therefore, any reduction in opioid consumption is directly indicative of the analgesic potential of the regional block. Assessing the 24-hour total opioid consumption, we noted that there was a tendency for reduced morphine consumption with ESPB as compared to control with an overall reduction of 35 mg. However, the CI was wide-ranging from − 77.01 to 6.52, turning the results non-significant. It can be noted from the forest plot that of the four studies comparing ESPB vs. control, the studies of Kim et al [15] and Mostafa et al [20] found limited or no difference in total opioid consumption while the remaining studies noted a significantly large reduction of opioid consumption. This could be because the procedures were minimally invasive (percutaneous and laparoscopic) in the former studies resulting in limited pain which was easily managed by the standardized analgesic protocol and ESPB had little additive effect. The review also did not find any significant difference in opioid consumption between ESPB and spinal analgesia suggesting that there may be equivalence between the two regional analgesic modalities. However, of the three studies in this comparison, two used intrathecal morphine while one used epidural block. The former studies noted better outcomes with intrathecal morphine while Zubair et al [16] found ESPB to be better than epidural analgesia. The primary disadvantage of spinal analgesia is its associated complications like headaches, respiratory depression, hypotension, backache, etc [27]. However, these could not be compared in a meta-analysis owing to a limited sample size of the RCTs resulting in too few complications. Given the differences among studies and limited data, further trials comparing ESPB with spinal analgesic techniques are needed to demonstrate equivalence between the two.

In the second part of the meta-analysis, we noted only a minimal reduction of pain scores with ESPB vs. control, and that too only at 12 and 24 h. The reduction of pain on a ten-point scale was only 0.41 and 0.11 respectively. The results might have been statistically significant but would not qualify for the minimum clinically important difference which is considered worthwhile by the patient [28]. On the other hand, the meta-analysis also noted no significant difference in pain scores between ESPB and spinal analgesia at all time points. Given the lack of difference in pain scores, it is necessary to distinguish between studies which used ESPB as a single shot or as continuous blocks, as the latter would produce a longer effect. However, the two studies using continuous blocks reported conflicting evidence. Kang et al [21] compared programmed intermittent bolus injections of ESPB with intrathecal morphine and found no difference in 48 h opioid consumption between the two techniques. On the other hand, Zubair et al [16] noted that continuous ESPB provided superior pain control as compared to thoracic epidural analgesia. Due to the scarcity of data, further trials are needed to confirm if continuous ESPB results in better outcomes as compared to single shot blocks.

The only adverse event which could be quantitatively examined in the meta-analysis was PONV. Given the tendency of reduced opioid consumption with ESPB vs. control, one may expect a significantly reduced incidence of PONV with ESPB. However, there was no difference in the risk of PONV between ESPB vs. control. Limited data from two trials found no difference in the risk of PONV between ESPB and spinal analgesia as well. Furthermore, none of the trials reported any major complications with the use of ESPB. No patient had local anesthetic toxicity, nerve injury, pneumothorax, or vascular injury in the ESPB group. This could be credited to the safety of ESPB where the needle penetration path and position are away from major neurovascular structures [29].

The results of our review are similar to the past meta-analysis of Bhushan et al [10] wherein they too did not find any significant analgesic effect of ESPB for liver surgeries. However, their study could include only six RCTs and the authors also included a comparison of ESPB with other blocks like quadratus lumborum block. Inclusion of a mix of placebo, spinal and other blocks in the control group results in biased evidence decreasing the credibility of the results. In the current review, we excluded comparisons with other regional blocks, updated the literature search and included four more RCTs, and also conducted a separate analysis of ESPB vs. control and ESPB vs. spinal analgesia to provide high-quality evidence on the subject.

The lack of effectiveness of ESPB in liver surgery could be related to the anatomy of the block. The ESPB is primarily a paraspinal fascial plane block wherein the local anesthetic is injected between the erector spinae muscle and the thoracic transverse processes. It predominantly blocks the posterior rami of the thoracic and abdominal spinal nerves with little effect on the anterior rami resulting in minimal analgesia beyond the mid-axillary line [4]. While the thoracic epidural is technically more difficult, it may still be the preferred approach in patients undergoing liver surgeries.

There are limitations to this meta-analysis. The primary drawback is the extremely high heterogeneity noted in the analysis. Indeed, despite including a very specific cohort of liver surgery patients, there were several methodological differences in the included studies. Variations in the type of liver surgery, invasiveness of the procedure, type of local anesthetic, its concentration and volume, the level of the injection, type of drug in PCA, and postoperative standard analgesic protocol were noted among the studies which could have led to such high heterogeneity. Secondly, while most of the trials used single injections of ESPB, two of the studies used continuous blocks. Due to limited data, we could not differentiate the outcomes of single vs. continuous ESPB blocks. Thirdly, despite an updated literature search, only nine RCTs were available for the meta-analysis and most of them had a small sample size. Also, the division of studies based on the control group protocol further reduced the number of trials in each meta-analysis. Lastly, the trials were from a few specific countries and the results should be generalized with caution.

Our results have clinical significance. Based on current evidence, routine use of ESPB cannot be recommended for patients undergoing liver surgeries. Secondly, as nursing personnel are closely involved in the perioperative and postoperative management of patients, patients receiving ESPB should not be deprioritized during control of postoperative pain. Nursing personnel should maintain a high index of suspicion even for those receiving ESPB till further evidence establishes the efficacy of this block in liver surgery patients.

## Conclusions

Based on currently available evidence, ESPB may not provide any significant postoperative analgesia in patients undergoing liver surgeries. There was a tendency of reduced opioid consumption with ESPB. Limited data also showed that ESPB and spinal analgesia had no difference in pain scores and 24-hour analgesic consumption.

### Electronic supplementary material

Below is the link to the electronic supplementary material.

Supplementary Material 1

Supplementary Material 2

Supplementary Material 3

## Data Availability

The authors confirm that the data supporting the findings of this study are available within the article and in its supplementary materials.

## References

[1] Tzimas P, Prout J, Papadopoulos G, Mallett SV. Epidural anaesthesia and analgesia for liver resection. Anaesthesia. 2013;68:628–35. 10.1111/ANAE.12191. 23662750 10.1111/ANAE.12191

[2] Agarwal V, Divatia JV. Enhanced recovery after surgery in liver resection: current concepts and controversies. Korean J Anesthesiol. 2019;72:119–29. 10.4097/KJA.D.19.00010. 30841029 10.4097/KJA.D.19.00010 PMC6458514

[3] Pirie K, Traer E, Finniss D, Myles PS, Riedel B. Current approaches to acute postoperative pain management after major abdominal surgery: a narrative review and future directions. Br J Anaesth. 2022;129:378–93. 10.1016/J.BJA.2022.05.029. 35803751 10.1016/J.BJA.2022.05.029

[4] Forero M, Adhikary SD, Lopez H, Tsui C, Chin KJ. The Erector Spinae Plane Block: a novel analgesic technique in thoracic neuropathic Pain. Reg Anesth Pain Med. 2016;41:621–7. 10.1097/AAP.0000000000000451. 27501016 10.1097/AAP.0000000000000451

[5] Chin KJ, El-Boghdadly K. Mechanisms of action of the erector spinae plane (ESP) block: a narrative review. Can J Anaesth. 2021;68:387–408. 10.1007/S12630-020-01875-2. 33403545 10.1007/S12630-020-01875-2

[6] De Cassai A, Bonanno C, Padrini R, Geraldini F, Boscolo A, Navalesi P, et al. Pharmacokinetics of lidocaine after bilateral ESP block. Reg Anesth Pain Med. 2021;46:86–9. 10.1136/RAPM-2020-101718. 32868484 10.1136/RAPM-2020-101718

[7] Leong RW, Tan ESJ, Wong SN, Tan KH, Liu CW. Efficacy of erector spinae plane block for analgesia in breast surgery: a systematic review and meta-analysis. Anaesthesia. 2021;76:404–13. 10.1111/ANAE.15164. 32609389 10.1111/ANAE.15164

[8] Koo CH, Lee HT, Na HS, Ryu JH, Shin HJ. Efficacy of Erector Spinae Plane Block for Analgesia in thoracic surgery: a systematic review and Meta-analysis. J Cardiothorac Vasc Anesth. 2022;36:1387–95. 10.1053/J.JVCA.2021.06.029. 34301447 10.1053/J.JVCA.2021.06.029

[9] Viderman D, Aubakirova M, Abdildin YG. Erector Spinae Plane Block in Abdominal surgery: a Meta-analysis. Front Med. 2022;9. 10.3389/FMED.2022.812531. 10.3389/fmed.2022.812531 PMC8904394 35280917

[10] Bhushan S, Huang X, Su X, Luo L, Xiao Z. Ultrasound-guided erector spinae plane block for postoperative analgesia in patients after liver surgery: a systematic review and meta-analysis on randomized comparative studies. Int J Surg. 2022;103. 10.1016/J.IJSU.2022.106689. 10.1016/j.ijsu.2022.106689 35662584

[11] Higgins J, Thomas J, Chandler J, Cumpston M, Li T, Page M, et al. Cochrane Handbook for Systematic Reviews of Interventions. Version 6. Cochrane; 2019. 10.1002/9781119536604. 10.1002/14651858.ED000142 PMC10284251 31643080

[12] Wan X, Wang W, Liu J, Tong T. Estimating the sample mean and standard deviation from the sample size, median, range and/or interquartile range. BMC Med Res Methodol. 2014;14:135. 10.1186/1471-2288-14-135. 25524443 10.1186/1471-2288-14-135 PMC4383202

[13] Opioid (Opiate). Equianalgesia Conversion Calculator - ClinCalc.com. https://clincalc.com/Opioids/. Accessed 30 Apr 2023.

[14] Page MJ, McKenzie JE, Bossuyt PM, Boutron I, Hoffmann TC, Mulrow CD, et al. The PRISMA 2020 statement: an updated guideline for reporting systematic reviews. Int J Surg. 2021;88:105906. 10.1016/j.ijsu.2021.105906. 33789826 10.1016/j.ijsu.2021.105906

[15] Kim D, Kim JM, Choi GS, Heo G, Kim GS, Jeong JS. Ultrasound-guided erector spinae plane block for postoperative analgesia in laparoscopic liver resection: a prospective, randomised controlled, patient and observer-blinded study. Eur J Anaesthesiol. 2021;38(Suppl 2):S106–12. 10.1097/EJA.0000000000001475. 33653982 10.1097/EJA.0000000000001475

[16] Zubair M, Adil Khan M, Khan MNA, Iqbal S, Ashraf M, Saleem SA. Comparison of continuous thoracic epidural with Erector Spinae Block for Postoperative Analgesia in Adult Living Donor Hepatectomy. Cureus. 2022;14. 10.7759/CUREUS.23151. 10.7759/cureus.23151 PMC9010007 35444875

[17] Hacıbeyoğlu G, Topal A, Küçükkartallar T, Yılmaz R, Arıcan Ş, Uzun ST. Investigation of the effect of ultrasonography-guided bilateral erector spinae plane block on postoperative opioid consumption and pain scores in patients undergoing hepatectomy: a prospective, randomized, controlled study. Sao Paulo Med J. 2022;140:144–52. 10.1590/1516-3180.2020.0757.R1.08062021. 35043869 10.1590/1516-3180.2020.0757.R1.08062021 PMC9623837

[18] Huang X, Wang J, Zhang J, Kang Y, Sandeep B, Yang J. Ultrasound-guided erector spinae plane block improves analgesia after laparoscopic hepatectomy: a randomised controlled trial. Br J Anaesth. 2022;129:445–53. 10.1016/J.BJA.2022.05.013. 35803754 10.1016/J.BJA.2022.05.013

[19] Elshafie MA, Khalil MK, Elsheikh ML, Mowafy NI. Erector Spinae Block with Opioid Free Anesthesia in Cirrhotic patients undergoing hepatic resection: a Randomized Controlled Trial. Local Reg Anesth. 2022;15:1–10. 10.2147/LRA.S343347. 35115825 10.2147/LRA.S343347 PMC8801329

[20] Mostafa SF, El Mourad MB. Ultrasound guided erector spinae plane block for percutaneous radiofrequency ablation of liver tumors. https://doi.org/101080/1110184920201854156. 2020;36:305–11. 10.1080/11101849.2020.1854156.

[21] Kang RA, Chin KJ, Kim GS, Gwak MS, Kim JM, Choi GS, et al. Bilateral continuous erector spinae plane block using a programmed intermittent bolus regimen versus intrathecal morphine for postoperative analgesia in living donor laparoscopic hepatectomy: a randomized controlled trial. J Clin Anesth. 2021;75. 10.1016/J.JCLINANE.2021.110479. 10.1016/j.jclinane.2021.110479 34455152

[22] Fu J, Zhang G, Qiu Y. Erector Spinae plane block for postoperative pain and recovery in hepatectomy: a randomized controlled trial. Med (Baltim). 2020;99:e22251. 10.1097/MD.0000000000022251. 10.1097/MD.0000000000022251 PMC10545310 33031265

[23] Kang RA, Chin KJ, Gwak MS, Kim GS, Choi SJ, Kim JM, et al. Bilateral single-injection erector spinae plane block versus intrathecal morphine for postoperative analgesia in living donor laparoscopic hepatectomy: a randomized non-inferiority trial. Reg Anesth Pain Med. 2019;44:1059–65. 10.1136/RAPM-2019-100902. 10.1136/RAPM-2019-100902 31649028

[24] Chin KJ, Versyck B, Elsharkawy H, Rojas Gomez MF, Sala-Blanch X, Reina MA. Anatomical basis of fascial plane blocks. Reg Anesth Pain Med. 2021;46:581–99. 10.1136/RAPM-2021-102506. 34145071 10.1136/RAPM-2021-102506

[25] Oh SK, Lim BG, Won YJ, Lee DK, Kim SS. Analgesic efficacy of erector spinae plane block in lumbar spine surgery: a systematic review and meta-analysis. J Clin Anesth. 2022;78. 10.1016/J.JCLINANE.2022.110647. 10.1016/j.jclinane.2022.110647 35030493

[26] Frassanito L, Zanfini BA, Catarci S, Sonnino C, Giuri PP, Draisci G. Erector Spinae plane block for postoperative analgesia after total laparoscopic hysterectomy: case series and review of the literature. Eur Rev Med Pharmacol Sci. 2020;24:3892–7. 10.26355/EURREV_202004_20855. 32329864 10.26355/EURREV_202004_20855

[27] Vadhanan P. Recent updates in spinal Anesthesia-A narrative review. Asian J Anesthesiol. 2021;59:41–50. 10.6859/AJA.202106_59(2. 33951783 10.6859/AJA.202106_59(2

[28] Copay AG, Subach BR, Glassman SD, Polly DW, Schuler TC. Understanding the minimum clinically important difference: a review of concepts and methods. Spine J. 2007;7:541–6. 17448732 10.1016/j.spinee.2007.01.008

[29] Kot P, Rodriguez P, Granell M, Cano B, Rovira L, Morales J, et al. The erector spinae plane block: a narrative review. Korean J Anesthesiol. 2019;72:209–20. 10.4097/KJA.D.19.00012. 30886130 10.4097/KJA.D.19.00012 PMC6547235

