# Supplementary material for: Efficacy of erector spinae plane block for postoperative analgesia after liver surgeries: a systematic review and meta-analysis
Source: BMC Anesthesiol. 2024 Jul 20;24:246. doi: 10.1186/s12871-024-02635-1 (PMC11264924; doi:10.1186/s12871-024-02635-1)
Supplement: Supplementary file 1 — Supplementary Material 1 [file 12871_2024_2635_MOESM1_ESM.docx]

Supplementary Table 1. Search strategy

| Keywords | Details |
| --- | --- |
| (abdominal surgery) AND (erector spinae plane block) | ("abdom surg"[Journal] OR ("abdominal"[All Fields] AND "surgery"[All Fields]) OR "abdominal surgery"[All Fields]) AND (("erector"[All Fields] OR "erectores"[All Fields] OR "erectors"[All Fields]) AND "spinae"[All Fields] AND ("aircraft"[MeSH Terms] OR "aircraft"[All Fields] OR "plane"[All Fields] OR "planes"[All Fields]) AND ("block"[All Fields] OR "blocked"[All Fields] OR "blocking"[All Fields] OR "blockings"[All Fields] OR "blocks"[All Fields])) |
| (liver surgery) AND (erector spinae plane block) | ("liver"[MeSH Terms] OR "liver"[All Fields] OR "livers"[All Fields] OR "liver s"[All Fields]) AND ("surgery"[MeSH Subheading] OR "surgery"[All Fields] OR "surgical procedures, operative"[MeSH Terms] OR ("surgical"[All Fields] AND "procedures"[All Fields] AND "operative"[All Fields]) OR "operative surgical procedures"[All Fields] OR "general surgery"[MeSH Terms] OR ("general"[All Fields] AND "surgery"[All Fields]) OR "general surgery"[All Fields] OR "surgery s"[All Fields] OR "surgerys"[All Fields] OR "surgeries"[All Fields]) AND (("erector"[All Fields] OR "erectores"[All Fields] OR "erectors"[All Fields]) AND "spinae"[All Fields] AND ("aircraft"[MeSH Terms] OR "aircraft"[All Fields] OR "plane"[All Fields] OR "planes"[All Fields]) AND ("block"[All Fields] OR "blocked"[All Fields] OR "blocking"[All Fields] OR "blockings"[All Fields] OR "blocks"[All Fields])) |
| ((hepatic) OR (hepatectomy)) AND (erector spinae plane block) | ("hepatic"[All Fields] OR "hepatophyta"[MeSH Terms] OR "hepatophyta"[All Fields] OR "hepatics"[All Fields] OR ("hepatectomy"[MeSH Terms] OR "hepatectomy"[All Fields] OR "hepatectomies"[All Fields])) AND (("erector"[All Fields] OR "erectores"[All Fields] OR "erectors"[All Fields]) AND "spinae"[All Fields] AND ("aircraft"[MeSH Terms] OR "aircraft"[All Fields] OR "plane"[All Fields] OR "planes"[All Fields]) AND ("block"[All Fields] OR "blocked"[All Fields] OR "blocking"[All Fields] OR "blockings"[All Fields] OR "blocks"[All Fields])) |
