# Supplementary material for: Efficacy of erector spinae plane block for postoperative analgesia after liver surgeries: a systematic review and meta-analysis
Source: BMC Anesthesiol. 2024 Jul 20;24:246. doi: 10.1186/s12871-024-02635-1 (PMC11264924; doi:10.1186/s12871-024-02635-1)
Supplement: Supplementary file 2 — Supplementary Material 2 [file 12871_2024_2635_MOESM2_ESM.docx]

Supplementary Table 2. Risk of bias analysis

| Study | Randomization process | Deviation from intended intervention | Missing outcome data | Measurement of outcomes | Selection of reported result | Overall risk of bias |
| --- | --- | --- | --- | --- | --- | --- |
| Kang 2019[23] | Low risk | Low risk | Low risk | Low risk | Low risk | Low risk |
| Fu 2020[22] | Some concerns | Some concerns | Low risk | High risk | Low risk | High risk |
| Mostafa 2020[20] | Low risk | Low risk | Low risk | Some concerns | Low risk | Some concerns |
| Kang 2021[21] | Low risk | Low risk | Low risk | Low risk | Low risk | Low risk |
| Kim 2021[15] | Low risk | Low risk | Low risk | Low risk | Low risk | Low risk |
| Elshafie 2022[19] | Low risk | Low risk | Low risk | Low risk | Low risk | Low risk |
| Hacıbeyoğlu 2022[17] | Low risk | Low risk | Low risk | Low risk | Low risk | Low risk |
| Huang 2022[18] | Low risk | Low risk | Low risk | Low risk | Low risk | Low risk |
| Zubair 2022[16] | Low risk | Low risk | Low risk | Low risk | Low risk | Low risk |
