# Supplementary material for: Efficacy of erector spinae plane block for postoperative analgesia after liver surgeries: a systematic review and meta-analysis
Source: BMC Anesthesiol. 2024 Jul 20;24:246. doi: 10.1186/s12871-024-02635-1 (PMC11264924; doi:10.1186/s12871-024-02635-1)
Supplement: Supplementary file 3 — Supplementary Material 3 [file 12871_2024_2635_MOESM3_ESM.docx]

| Supplementary Table 3. GRADE assessment of evidence | | | | | | | | | | | |
| --- | --- | --- | --- | --- | --- | --- | --- | --- | --- | --- | --- |
| **Certainty assessment** | | | | | | | **Summary of findings** | | | | |
| **Participants (studies) Follow-up** | **Risk of bias** | **Inconsistency** | **Indirectness** | **Imprecision** | **Publication bias** | **Overall certainty of evidence** | **Study event rates (%)** | | **Relative effect (95% CI)** | **Anticipated absolute effects** | |
|  |  |  |  |  |  |  | **With placebo** | **With ESPB** |  | **Risk with placebo** | **Risk difference with ESPB** |
| **Total opioid consumption - ESPB vs no/sham block** | | | | | | | | | | | |
| 220 (4 RCTs) | not serious | not serious | not serious | serious^a^ | none | ⨁⨁⨁◯ Moderate | 110 | 110 | - | The mean ESPB vs no/sham block was **0** | MD **35.25 lower** (77.01 lower to 6.52 higher) |
| **Total opioid consumption - ESPB vs spinal analgesia** | | | | | | | | | | | |
| 153 (3 RCTs) | not serious | not serious | not serious | serious^a^ | none | ⨁⨁⨁◯ Moderate | 77 | 76 | - | The mean ESPB vs spinal was **0** | MD **2.32 higher** (6.12 lower to 10.77 higher) |
| **Pain score ESPB vs control - 6-8 hours** | | | | | | | | | | | |
| 240 (4 RCTs) | serious^b^ | not serious | not serious | not serious | none | ⨁⨁⨁◯ Moderate | 120 | 120 | - | The mean ESPB vs control - 6-8 hours was **0** | MD **0.66 lower** (1.48 lower to 0.16 higher) |
| **Pain score ESPB vs control - 12 hours** | | | | | | | | | | | |
| 180 (3 RCTs) | serious^b^ | not serious | not serious | not serious | none | ⨁⨁⨁◯ Moderate | 90 | 90 | - | The mean ESPB vs control - 12 hours was **0** | MD **0.41 lower** (0.76 lower to 0.05 lower) |
| **Pain score ESPB vs control - 24 hours** | | | | | | | | | | | |
| 240 (4 RCTs) | serious^b^ | not serious | not serious | not serious | none | ⨁⨁⨁◯ Moderate | 120 | 120 | - | The mean ESPB vs control - 24 hours was **0** | MD **0.26 lower** (1.14 lower to 0.62 higher) |
| **Pain score ESPB vs control - 48 hours** | | | | | | | | | | | |
| 180 (3 RCTs) | serious^b^ | not serious | not serious | not serious | none | ⨁⨁⨁◯ Moderate | 90 | 90 | - | The mean ESPB vs control - 48 hours was **0** | MD **0.11 lower** (0.2 lower to 0.02 lower) |
| **Pain score ESPB vs spinal - 6-8 hours** | | | | | | | | | | | |
| 153 (3 RCTs) | not serious | not serious | not serious | serious^c^ | none | ⨁⨁⨁◯ Moderate | 77 | 76 | - | The mean ESPB vs spinal - 6-8 hours was **0** | MD **0.37 higher** (0.95 lower to 1.69 higher) |
| **Pain score ESPB vs spinal - 24 hours** | | | | | | | | | | | |
| 153 (3 RCTs) | not serious | not serious | not serious | serious^c^ | none | ⨁⨁⨁◯ Moderate | 77 | 76 | - | The mean ESPB vs spinal - 24 hours was **0** | MD **0.23 higher** (0.58 lower to 1.04 higher) |
| **Pain score ESPB vs spinal - 48 hours** | | | | | | | | | | | |
| 153 (3 RCTs) | not serious | not serious | not serious | serious^c^ | none | ⨁⨁⨁◯ Moderate | 77 | 76 | - | The mean ESPB vs spinal - 48 hours was **0** | MD **0.75 lower** (1.89 lower to 0.4 higher) |
| **PONV - ESPB vs no/sham block** | | | | | | | | | | | |
| 330 (6 RCTs) | serious^b^ | not serious | not serious | not serious | none | ⨁⨁⨁◯ Moderate | 60/165 (36.4%) | 41/165 (24.8%) | **RR 0.70** (0.37 to 1.33) | 364 per 1,000 | **109 fewer per 1,000** (from 229 fewer to 120 more) |
| **PONV - ESPB vs spinal analgesia** | | | | | | | | | | | |
| 113 (2 RCTs) | not serious | not serious | not serious | serious^c^ | none | ⨁⨁⨁◯ Moderate | 41/57 (71.9%) | 21/56 (37.5%) | **RR 0.53** (0.27 to 1.06) | 719 per 1,000 | **338 fewer per 1,000** (from 525 fewer to 43 more) |

**CI:** confidence interval; **MD:** mean difference; **RR:** risk ratio

#### Explanations

a. Limited sample size of included studies with variation in results

b. high risk of bias in Fu et al and some concerns in Mostafa et al

c. limited number of studies
